# Improvement of Adipose Macrophage Polarization in High Fat Diet-Induced Obese GHSR Knockout Mice

**DOI:** 10.1155/2018/4924325

**Published:** 2018-07-10

**Authors:** Fang Yuan, Jian Ma, Xinxin Xiang, He Lan, Yanhui Xu, Jing Zhao, Yin Li, Weizhen Zhang

**Affiliations:** ^1^Department of Physiology and Pathophysiology, Peking University Health Science Center; Key Laboratory of Molecular Cardiovascular Science, Ministry of Education, Beijing 100191, China; ^2^Department of Geriatrics, No. 401 Hospital of PLA, Qingdao 266071, China; ^3^Department of Pathology, Central Hospital of Zibo, Zibo 255000, China; ^4^Department of Clinical Laboratory, Xuanwu Hospital, Capital Medical University, Beijing 100053, China; ^5^Department of Surgery, University of Michigan, Ann Arbor, MI, USA

## Abstract

**Purpose:**

Adipose tissue inflammation is the key linking obesity to insulin resistance. Over 50% of the interstitial cells in adipose tissue are macrophages, which produce inflammatory cytokines and therefore play an important role in the progression of insulin resistance. Within this classification view, macrophage biology is driven by two polarization phenotypes, M_1_ (proinflammatory) and M_2_ (anti-inflammatory). The unique functional receptor of ghrelin, growth hormone secretagogue receptor (GHSR), is a classic seven-transmembrane G protein-coupled receptor that is linked to multiple intracellular signaling pathways. Knockout of GHSR improves the obesity and glucose metabolic disorders, suggesting a crucial role of ghrelin activity in insulin resistance. Here, we discussed whether macrophage polarization phenotypes in adipose tissue were changed in GHSR knockout (GHSR-/-) mice.

**Methods:**

GHSR-/- mice were fed with normal chow diet (NCD) or high fat diet (HFD). Markers of different macrophage polarization phenotypes were detected by real-time RT-PCR.

**Results:**

The size of adipocytes decreased and interstitial cells, especially infiltrated macrophages, reduced in epididymal adipose tissue of GHSR-/- mice fed with HFD. Compared with wild type mice, the mRNA levels of inflammatory adipokines such as resistin, IL-6, and PAI-1 were significantly lower in epididymal adipose tissue of GHSR-/- mice, whereas anti-inflammatory adipokine, adiponectin, was significantly higher. M_1_ markers, MCP-1, TNF-*α*, and iNOS, were significantly lower in epididymal adipose tissue of GHSR-/- mice, whereas M_2_ markers, Arg-1, Mgl-1, were Mrc1, were significantly higher.

**Conclusion:**

The GHSR-/- mice fed with HFD showed suppressed adipose inflammation, reduced macrophage infiltration, and enhanced M_2_ polarization of macrophages in adipose tissue, which improved insulin sensitivity.

## 1. Introduction

As the natural ligand of the orphan growth hormone secretagogue receptor type 1a (GHSR1a) [1, 2], ghrelin is a 28-amino acid peptide which is secreted by gastric oxyntic glands and octanoylated by ghrelin O-acyl transferase (GOAT), the only enzyme that has been identified [3–5], which is expressed predominantly in the stomach, gut, and pancreas and also at other sites [6]. Human ghrelin mRNA codes for a 117 amino acid peptide, preproghrelin [7], which undergoes endoproteolytic processing and posttranslational modification to produce acyl ghrelin, des-acyl ghrelin, and obestatin [8–12]. Serum acyl ghrelin levels increase with fasting, suggesting that it is an orexigenic hormone involved in meal initiation [13, 14]. Acyl ghrelin regulates somatic growth, feeding behavior, and energy homeostasis in mammals [15, 16], and it also stimulates motility of the stomach and small intestine and acid secretion in rats [4, 17]. In our previous study, we found that GHSR knockout improved glucose metabolism in skeletal muscle in mice [18].

Insulin resistance is the common basis in pathogenesis for obesity-related diseases [19], such as metabolic syndrome, type 2 diabetes, and atherosclerosis, which are low-grade aspecific proinflammatory states. Reciprocal causation of insulin resistance, obesity, and inflammation indicates that inflammatory factors, chemokines, and immunocytes could reduce insulin sensitivity directly or indirectly, participating in the occurrence and development of insulin resistance.

As immune cells, macrophages are pluripotent and play an important role in innate immunity. Macrophage polarization is considered as phenotypic heterogeneity of the mononuclear phagocyte system [20]. As a result of cellular differentiation, widespread tissue distribution, and responsiveness to many endogenous and exogenous stimuli, macrophages are divided into classical or M_1_ and alternative or M_2_ polarization. M_1_ macrophages are also named proinflammatory macrophages, and M_2_ macrophages are named anti-inflammatory macrophages; the former secret proinflammatory factors and have proinflammatory functions, and the later have anti-inflammatory functions and are involved in tissue repair [21].

During obesity, the macrophage infiltration in adipose tissue is enhanced, and mainly infiltrated phenotype is M_1_ [21]. The interaction between M_1_ macrophages and adipocytes promotes the secretion of inflammatory factors, which results in the insulin resistance. Suppression of inflammation or inducing transformation of macrophages in adipose tissue from M_1_ to M_2_ can relieve insulin resistance and improve related metabolic syndrome.

In the present study, we investigated the macrophage polarization in the adipose tissue of GHSR knockout mice and contributed to a new mechanism of GHSR knockout-improved insulin sensitivity.

## 2. Materials and Methods

### 2.1. Materials

Acyl ghrelin was purchased from Phoenix Pharmaceuticals Inc. (Burlingame, CA). Rabbit anti-phospho-AKT (Ser473) and AKT antibodies were from Cell Signaling Technology (Beverly, MA). Rat anti-LAMP-2 (Mac3/84) antibody and chicken anti-rabbit fluorescein isothiocyanate-conjugated IgG were from Santa Cruz Biotechnology Inc. (Santa Cruz, CA). DyLigh 594-goat anti-rat IgG was from EarthOx LLC (EarthOx, CA).

### 2.2. Ethical Approval

The animals used in this study were handled in accordance with the Guide for the Care and Use of Laboratory Animals published by the US National Institutes of Health (NIH publication no. 85-23, revised 1996), and all the experimental protocols were approved by the Animal Care and Use Committee of Peking University.

### 2.3. Animals and Treatments

C57BL/6J mice,* Ghsr1a *knockout (GHSR-/-) mice, were used in the present study.* Ghsr1a* gene knockout mice in which exon 1 and exon 2 had been deleted were obtained from the Shanghai Research Center for Biomodel Organisms (Shanghai, China) [18, 22]. Deletion of the* Ghsr1a* gene fragment was confirmed by the absence of relative gene products examined by RT-PCR. 6-week-old male mice were housed in specific pathogen-free microisolators and maintained in a regulated environment (24°C, 12h light-dark cycle, with lights on at 07:00 AM). Regular chow and water were available* ad libitum*. Mice were assigned to receive standard laboratory chow (NCD) or a high fat diet (HFD) (45% fat, D12451; Research Diets, New Brunswick, NJ, USA) for 12 weeks.

### 2.4. Cell Culture

RAW264.7 cells, the murine peritoneal macrophage-like cell line, were cultured in growth medium (high-glucose Dulbecco's modified Eagle's medium (DMEM); Invitrogen, Grand Island, NY, USA) supplemented with 10% heat-inactivated fetal bovine serum (FBS; US Biotechnologies), 100 units/ml penicillin, and 100 units/ml streptomycin (Invitrogen) and incubated at 37°C with 5% CO_2_. Cells were passaged weekly after trypsin-EDTA detachment. All studies were performed on RAW264.7 cells at passages 20-25. Then cells were cultured with LPS (10 ng/ml) or IL-4 (10 ng/ml) to generate classically (M_1_) or alternatively (M_2_) polarized cells.

### 2.5. Glucose Tolerance Test

Glucose tolerance test was performed as described [18]. Blood was drawn from a cut at the tip of the tail at 0, 30, 60, 90, and 120 min, and glucose concentrations were detected immediately with Glucotrend from Roche Diagnostics (Mannheim, Germany) according to the manufacturer's instructions.

### 2.6. RNA Extraction and Quantitative Real-Time PCR Analysis

Total RNA was isolated using the Trizol reagent. Reverse transcription and quantitative real-time PCR were performed as previously described [23–25]. PCR was conducted in a 25*μ*l volume containing 2.5 ng cDNA, 5mM MgCl_2_, 0.2 mM dNTPs, 0.2*μ*M each primer, 1.25 U AmpliTaq Polymerase, and 1*μ*l 800x diluted SYBRGreen I stock using the Mx3000 multiplex quantitative PCR system (Stratagene, La Jolla, CA). The PCR program was as follows: holding 95°C for 7 min; 95°C for 30 s, 60°C for 35 s, and 72°C for 35 s. mRNA expression was quantified using the comparative cross threshold (CT) method. The CT value of the housekeeping gene *β*-actin was subtracted from the CT value of the target gene to obtain ^△^CT. The normalized fold changes of detected gene' mRNA expression were expressed as 2-^ΔΔCT^, where ΔΔCT = ΔCT sample - ΔCT control. PCR reactions were performed in duplicate and each experiment was repeated for 3-5 times. Primers used in this study were shown in Table 1.

### 2.7. Western Blot Analysis

As previously described [23–25], the adipose tissue and cultured cells were quickly harvested, rinsed thoroughly with PBS, and then homogenized on ice in lysis buffer (50 mM Tris-Cl, 15 mM EGTA, 100 mM NaCl, 0.1% Triton X-100 supplemented with protease inhibitor cocktail, pH 7.5). After centrifugation for 10 min at 4°C, the supernatant was used for western blot analysis. Protein concentration was measured by Bradford's method. A total of 60 *μ*g protein from each sample was loaded onto SDS-PAGE gel. Proteins were transferred to polyvinylidene fluoride membranes. The membranes were incubated for 1 h at room temperature with 5% fat-free milk in Tris buffered saline containing Tween-20, followed by incubation overnight at 4°C with the individual primary antibody. Specific reaction was detected using IRDye-conjugated second antibody and visualized using the Odyssey infrared imaging system (LI-COR Biosciences, Lincoln, NE). Quantification of image density in pixel was performed by using the Odyssey infrared imaging system (LI-COR Biosciences, Lincoln, NE).

### 2.8. H&E Histology

Mice were deeply anesthetized using pentobarbital. The epididymal adipose tissue was quickly removed and rinsed thoroughly with PBS. The tissue was fixed in 4% paraformaldehyde, dehydrated, embedded in wax, and sectioned at 6 *μ*m. Diameter of each adipocyte in each field was measured using image analysis software Image J (v1.48). For each group, cell sizes of about 450 adipocytes from 3-4 mice were measured and plotted as histograms.

### 2.9. Immunofluorescence

Mice were deeply anesthetized using pentobarbital. The same part of adipose tissue was quickly removed and rinsed thoroughly with PBS. The tissue was fixed in 4% paraformaldehyde, dehydrated, embedded in wax, and sectioned at 6*μ*m. Paraffin-embedded sections were dewaxed, rehydrated, and rinsed in PBS. After boiling for 10 min in 10 mM sodium citrate buffer (pH 6.0), sections were blocked in 1% BSA in PBS for 1 h at room temperature, then incubated overnight at 4°C with LAMP-2 (Mac3/84) antibody alone, and then washed in 1X PBS/0.1% Tween-20 three times for 5 minutes each. Tissue sections were then incubated at room temperature for 1 h with the following secondary antibodies (DyLigh 594-goat anti-rat IgG). Controls included substituting primary antibody with rat IgG. The nuclei were visualized by staining with Hoechst 33258 for 10 min. Photomicrographs were taken under a confocal laser-scanning microscope (Leica, Germany).

### 2.10. Statistical Analysis

Data were expressed as means±SEM. Data analysis used GraphPad Prism software. One-way ANOVA, Student-Newman-Keul's test (comparisons between multiple groups), or unpaired Student's* t*-test (between two groups) was used as appropriate. P value < 0.05 denotes statistical significance.

## 3. Results

### 3.1. Improvement of Glucose Metabolism in GHSR-/- Mice

**Figure 1:**
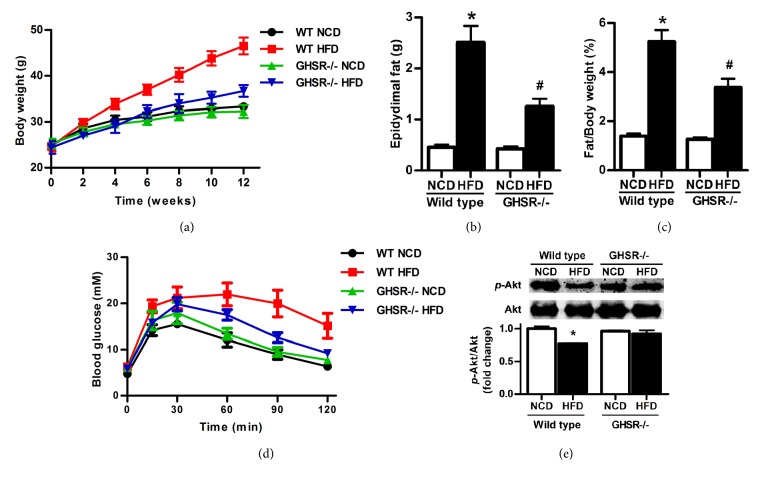
**Improvement of glucose metabolism in GHSR knockout mice**.** (a)** Body weight of GHSR knockout (GHSR-/-) mice and wild type (WT) mice fed with different diet.** (b)** The average daily food intake of GHSR-/- mice and wild type (WT) mice fed with normal chow diet or high fat diet for 12 weeks.** (c)** The average daily water intake of GHSR-/- mice and wild type mice fed with normal chow diet or high fat diet.** (d)** Results of glucose tolerance test using GHSR knockout mice and wild type mice fed with normal chow diet (NCD) or high fat diet (HFD) for 12 weeks. All results were expressed as means±SEM.** (e)** Western blotting results from GHSR knockout mice and wild type mice fed with normal chow diet or high fat diet for 12 weeks. Insulin at a dose of 2 IU/kg was injected intraperitoneally 15 min before harvest of the adipose tissue; phospho-Akt (Ser473) and Akt in adipose tissue were detected using specific antibodies. Total Akt level was used as internal control. Quantification of image analysis of* p*-Akt/Akt was expressed as means±SEM. *∗* denotes P<0.05 compared with wild type mice fed with NCD; ^**#**^ denotes P<0.05 compared with WT mice fed with HFD.

GHSR-/- mice and wild type (WT) mice were fed with normal chow diet (NCD) or high fat diet (HFD) separately. The food and water intake of mice were shown in Supplemental Figure 1. Food intake was less in mice fed the HF diet, especially in GHSR-/- mice. The body weight of wild type mice fed with HFD increased gradually in 4 weeks, while the GHSR-/- mice showed no significant differences in the body weight with different diet (Figure 1(a)). We also found that GHSR-/- mice showed less epididymal fat weight (Figure 1(b)) and fat/body weight ratio (Figure 1(c)) than WT mice when fed with HFD.

We further examined insulin sensitivity by performing glucose tolerance test. Wild type mice fed with high fat diet demonstrated severe hyperglycemia upon administration of glucose and impaired glucose tolerance, with typical glucose disposal curves of insulin resistance, whereas the glucose metabolism was not affected by high fat diet in GHSR-/- mice (Figure 1(d)). Insulin-induced Akt phosphorylation (Ser473) was significantly inhibited in wild type mice fed with HFD, but not in the GHSR-/- mice (Figure 1(e)). Therefore, deletion of GHSR could improve the glucose metabolic disorders induced by high fat diet.

### 3.2. Improvement of Adipose Tissue Inflammation in GHSR-/- Mice

**Figure 2:**
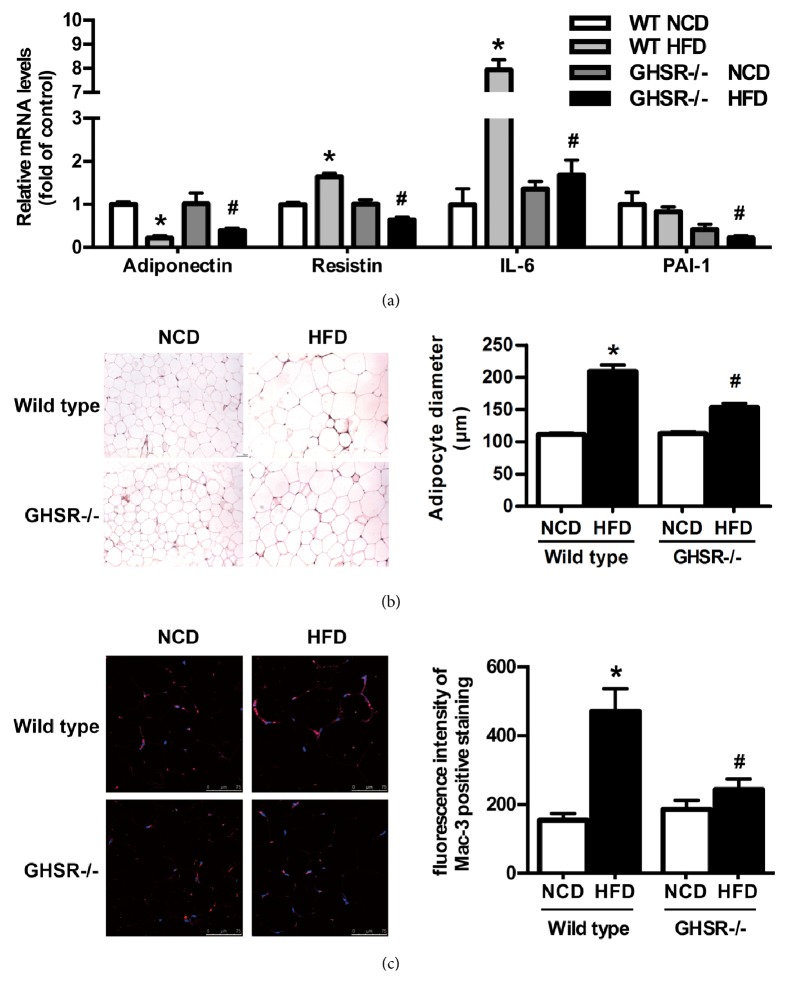
**Improvement of adipose tissue inflammation in GHSR knockout mice**.** (a)** Expression of inflammatory adipokines in epididymal adipose tissue of GHSR knockout mice, such as anti-inflammatory adipokine adiponectin and proinflammatory adipokines resistin, IL-6, and PAI-1. Relative mRNA levels were normalized to the levels for wild type mice fed with normal chow diet. Data are means±SEM, n=5.** (b)** Adipocytes volume and interstitial cell infiltration detected by H&E staining.** (c)** Macrophage infiltration detected by immunofluorescence histochemistry staining of Mac-3. *∗* denotes P<0.05 compared with WT mice fed with NCD; ^**#**^ denotes P<0.05 compared with WT mice fed with HFD.

It has been reported that insulin resistance and inflammation are intertwined and interdependent, whereas obesity is considered as a low-grade aspecific proinflammatory state. The above results indicated that obesity and glucose metabolic disorders induced by high fat diet were improved in GHSR-/- mice. We then detected the expression of inflammatory adipokines, such as resistin, interleukin-6 (IL-6), and Plasminogen activator inhibitor-1 (PAI-1), in epididymal adipose tissue. Compared with wild type mice fed with high fat diet, the mRNA levels of resistin and IL-6 were significantly lower in epididymal adipose tissues of GHSR-/- mice fed with high fat diet, whereas anti-inflammatory adipokine, adiponectin, was significantly higher (Figure 2(a)). H&E staining showed that the size of adipocytes decreased significantly and crown-like structure also reduced in epididymal adipose tissues of GHSR-/- mice fed with high fat diet (Figure 2(b)).

More than 50% of the interstitial cells in adipose tissue are macrophages, which are sensitive to the chemokines produced by adipose tissue under inflammatory state and can infiltrate in adipose tissue through chemotactic migration. Therefore, we then observed the variation of macrophages in epididymal adipose tissues of wild type mice and GHSR-/- mice by immunofluorescence. We found that Mac-3 staining, the marker of macrophages, significantly reduced in epididymal adipose tissues of GHSR-/- mice fed with high fat diet (Figure 2(c)), indicating the ameliorated macrophage infiltration and adipose inflammation.

### 3.3. Macrophage Polarization in Epididymal Adipose Tissue of GHSR-/- Mice

**Figure 3:**
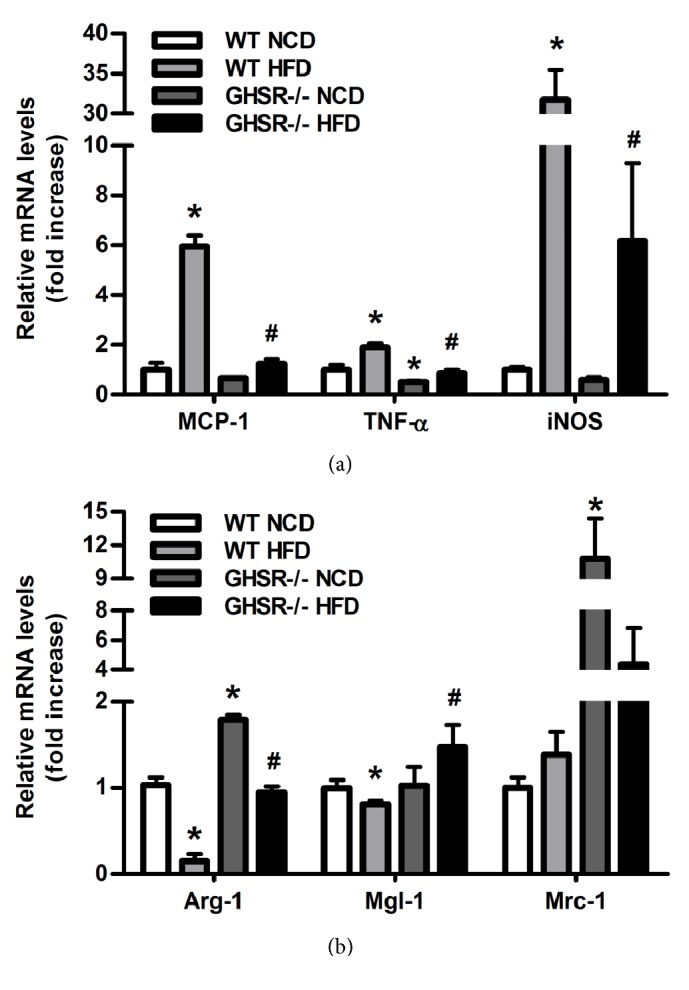
**Macrophage polarization in epididymal adipose tissue of GHSR knockout mice**. The mRNAs were extracted from the epididymal adipose tissue harvested from the GHSR knockout mice and wild type mice fed with different diet. Real-time PCR was performed to evaluate the expression of macrophage specific markers.** (a)** M_1_ markers MCP-1, TNF-*α*, iNOS and** (b)** M_2_ markers Arg-1, Mgl-1, Mrc1 were detected. Data are means±SEM n=5. *∗* denotes P<0.05 compared with WT mice fed with NCD; ^**#**^ denotes P<0.05 compared with WT mice fed with HFD.

Macrophage activation has been operationally defined as two antipodal polarization states, M_1_ (proinflammatory) and M_2_ (anti-inflammatory), which have different functions. We chose corresponding markers to detect the changes of macrophage polarization in epididymal adipose tissues of wild type mice and GHSR-/- mice with normal chow diet and high fat diet. We observed that, compared with normal chow diet, high fat diet significantly induced the mRNA levels of M_1_ markers, such as monocyte chemotactic protein-1 (MCP-1), tumor necrosis factor *α* (TNF-*α*), and inducible nitric oxide synthase (iNOS) in wild type mice (Figure 3(a)), while the M_2_ markers, like arginase-1 (Arg-1) and macrophage galactose-type lectin-1 (Mgl-1) (Figure 3(b)), decreased markedly, but mannose receptor C type 1 (Mrc1) remained unchanged. However, these trends were turned over in GHSR-/- mice (Figures 3(a) and 3(b)). All the results above indicated that deletion of GHSR could inhibit the adipose tissue inflammation, decrease the macrophage infiltration, and promote the macrophages polarization to M_2_.

### 3.4. Effect of Acyl Ghrelin on RAW264.7 Cells Polarization In Vitro

**Figure 4:**
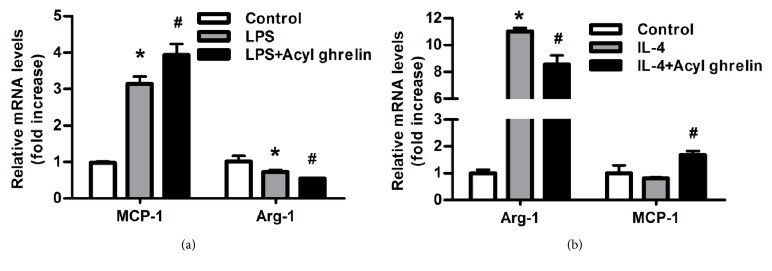
**Acyl ghrelin influences the effect of LPS or IL-4 on RAW264.7 cell polarization**. The mRNAs were extracted from the RAW 264.7 pretreated with acyl ghrelin for 30 minutes then for 6 hours with LPS (10 ng/ml)** (a)** or IL-4 (10 ng/ml)** (b)**. Real-time PCR was performed to evaluate the expression of macrophage specific markers Arg-1 and MCP-1. Data are means±SEM from 3 separate experiments. *∗* denotes P<0.05 compared with control group; ^**#**^ denotes P<0.05 compared with cells treated with LPS or IL-4 alone.

As reported, acyl ghrelin does its functions by binding with its receptor, GHSR1a. Therefore, the improvement of adipose tissue inflammation, decreased macrophage infiltration, and macrophages polarization in GHSR-/- mice might be due to the effect of acyl ghrelin. We then detected the direct effect of acyl ghrelin on the polarization of RAW264.7 murine macrophage cells, under the stimulation of LPS or IL-4, the classical polarizing inducer of M_1_ or M_2_. Arg-1 was chosen as M_2_ marker and MCP-1 was chosen as M_1_ marker. We found that acyl ghrelin could enhance the effect of LPS on increasing MCP-1 and decreasing Arg-1 mRNA levels (Figure 4(a)), while weakening the effect of IL-4 on increasing Arg-1 and decreasing MCP-1 mRNA levels (Figure 4(b)), which indicated the proinflammatory effect of acyl ghrelin.

These results suggest that acyl ghrelin could improve macrophage polarization under inflammatory state. Combined with the results* in vivo*, it can be concluded that deletion of GHSR would abolish the effect of acyl ghrelin, then inhibit the adipose tissue inflammation, decrease the macrophage infiltration, promote the macrophages polarization to M_2_, and finally improve insulin sensitivity and glucose metabolism.

## 4. Discussion

In the present study, we reported that GHSR knockout mice fed with HFD showed improved insulin sensitivity through improved adipose inflammation. This conclusion is supported by the following observations: (1) GHSR-/- mice fed with HFD showed suppressed expression of inflammatory adipokine in adipose tissue; (2) GHSR-/- mice fed with HFD showed reduced macrophage infiltration in adipose tissue; (3) GHSR-/- mice fed with HFD showed M2 polarization of macrophages in adipose tissue; (4) acyl ghrelin enhanced M_1_ polarization of RAW 264.7 macrophages under inflammatory status. To the best of our knowledge, this is the first report demonstrating the involvement of adipose-infiltrated macrophage polarization in the improvement of insulin sensitivity of GHSR knockout mice fed with HFD.

Ghrelin regulates processes associated with cancer, including cell proliferation, apoptosis, cell migration, cell invasion, inflammation, and angiogenesis [26]. Ghrelin also plays an important role in metabolism. It was reported that ghrelin could inhibit glucose-stimulated insulin secretion in a dose-dependent manner* in vitro* [27]; intravenous ghrelin injection decreased plasma insulin and increased plasma glucose levels, likely by inhibition of insulin secretion [27]; ablation of ghrelin, GHSR, or GOAT enhanced insulin release and attenuated impaired glucose tolerance in high fat diet-induced and leptin-deficient obese mice models [28]. Clinical studies also showed that infusing ghrelin to a supraphysiologic level would inhibit the glucose-stimulated insulin secretion and insulin sensitivity and would reduce glucose tolerance in humans [29]. In our studies, we also found that ablation of the action of acyl ghrelin by deletion of GHSR showed similar changes such as impaired insulin sensitivity, especially in high fat diet status.

Existing researches showed that, in the early stage of inflammatory diseases, the gradients of some inflammatory mediators play important roles in the recruitment of leukocytes to inflamed areas [30]. Different from the recruited neutrophils with short life spans due to apoptosis [31], macrophages, as immune cells, have longer life spans and play a more important role to clear neutrophils via phagocytosis. Opinions about the effect of ghrelin on inflammation are different; although some studies demonstrated it as a proinflammatory agent [32], most reports indicated that ghrelin acts as an anti-inflammatory factor [33, 34]. The reason might be the different cell lines and research objectives. In our study, we found that acyl ghrelin could promote the macrophages polarization to M_1_ directly* in vitro*, and knockout of its receptor GHSR1a inhibited adipose tissue inflammation, decreased the macrophage infiltration, and promoted the macrophages polarization to M_2_. Therefore, acyl ghrelin might exert proinflammatory effect, especially in our high fat diet system, which is consistent with the reports that ghrelin signaling has an important role in macrophage polarization and adipose tissue inflammation during aging [32] or feeding with high fructose corn syrup [35], in which the concurrences of obesity and insulin resistance are significantly greater. Since we have reported that the cytokine intermedin treatment inhibits chronic inflammation and improves systemic insulin sensitivity through restoring the M1/M2 balance in adipose tissues [36], the involvement of adipose-infiltrated macrophage polarization might be the key point in the improvement of insulin sensitivity of GHSR knockout mice fed with HFD.

Besides the direct effect of ghrelin elimination on macrophages polarization, the decrease of food intake in GHSR knockout mice should also be considered, which will in turn promote the resistance to high fat diet-induced obesity [37, 38] and adipose inflammation. However, in aging mice, knockout of GHSR did not affect the body weight and food intake [39], although adipose tissue inflammation and insulin resistance were improved [32]. Whether any other factors participate in the improvement of glucose tolerance and inflammatory status in GHSR knockout mice fed with HFD needs further studies.

In conclusion, our results indicated that acyl ghrelin could promote macrophage polarization to M_1_ under inflammatory state* in vitro*. While* in vivo*, deletion of GHSR would abolish the effect of acyl ghrelin and subsequently inhibit the adipose tissue inflammation, decrease the macrophage infiltration, promote the macrophages polarization to M_2_ phenotype, and finally improve insulin sensitivity. Our study will give a new insight about the function of acyl ghrelin and its receptor GHSR.

## Figures and Tables

**Table 1:** List and sequence of primers.

	Upstream primer (5′-3′)	Downstream primer (5′-3′)
Resistin	TCCTTGTCCCTGAACTGC	ACGAATGTCCCACGAGC
Adiponectin	ACCAGTATCAGGAAAAGAATGT	TAGAGAAGAAAGCCAGTAAATG
IL-6	AGTTGTGCAATGGCAATTCTG	GGAAATTGGGGTAGGAAGGAC
PAI-1	CCTCACCAACATCTTGGATGCT	TGCAGTGCCTGTGCTACAGAGA
MCP-1	ACTGAAGCCAGCTCTCTCTTCCTC	TTCCTTCTTGGGTCAGCACAGAC
TNF-*α*	CGTCGTAGCAAACCACCAAG	GAGATAGCAAATCGGCTGACG
iNOS	CCAAGCCCTCACCTACTTCC	CTCTGAGGGCTGACACAAGG
Arg-1	CTCCAAGCCAAAGTCCTTAGAG	AGGAGCTGTCATTAGGGACATC
Mgl-1	TGAGAAAGGCTTTAAGAACTGGG	GACCACCTGTAGTGATGTGGG
Mrc-1	AAACACAGACTGACCCTTCCC	GTTAGTGTACCGCACCCTCC
*β*-actin	ATCTGGCACCACACCTTC	AGCCAGGTCCAGACGCA

## Data Availability

The data used to support the findings of this study are available from the corresponding author upon request.

## Supplementary Materials

Supplementary Materials Online supplementary figure and legend were shown.
