# Supplementary material for: Improvement of Adipose Macrophage Polarization in High Fat Diet-Induced Obese GHSR Knockout Mice
Source: Biomed Res Int. 2018 Jul 10;2018:4924325. doi: 10.1155/2018/4924325 (PMC6077514; doi:10.1155/2018/4924325)

Online supplementary figure legend

Supplementary figure 1. General biological index of GHSR-/- mice

(A) The average daily food intake of GHSR-/- mice and wild type (WT) mice fed with normal chow diet (NCD) or high fat diet (HFD) for 12 weeks. **(B)** The average daily water intake of GHSR-/- mice and wild type mice fed with normal chow diet or high fat diet. All results were expressed as means±SEM. *denotes $P<0.05$ compared with wild type mice fed with NCD. [#] denotes $P<0.05$ compared with wild type mice fed with HFD.

Supplementary figure 1

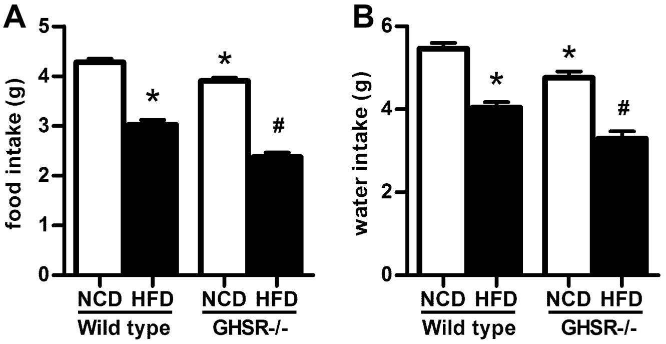

Supplement: Supplementary Materials — Online supplementary figure and legend were shown. [file 4924325.f1.pdf]
